# Detection rate and associated factors of thyroid nodules among residents in Qingdao, a coastal city in China

**DOI:** 10.1097/MD.0000000000049872

**Published:** 2026-07-24

**Authors:** Xuemei Sun, Yu Zhang, Pei Wang, Xiaopang Rao

**Affiliations:** a Department of Ultrasound, People’s Hospital of Chengyang District, Qingdao, Shandong, China; b Department of Endocrinology, People’s Hospital of Chengyang District, Qingdao, Shandong, China.

**Keywords:** cross-sectional study, detection rate, related factors, thyroid nodules

## Abstract

This study aims to investigate the detection rate and associated factors of thyroid nodules among a health checkup population in Qingdao, China, and provide a theoretical basis for intervention. Data for 15,283 healthy examinees, collected from January 2013 to December 2022, including demographic data, physical examinations, and thyroid ultrasonography, were used to determine the presence of thyroid nodules. The epidemiological characteristics and related risk factors of thyroid nodules were revealed through *t*-tests, chi-square tests, and logistic regression analysis. The survey showed that the detection rate of thyroid nodules among the examined population was 28.48%, with females having a significantly higher detection rate than males (35.51% vs 21.47%, *P* = .002). The detection rate of thyroid nodules increased with age. Factors such as high C-reactive protein (CRP), hyperglycemia, hypertension, and smoking history were significantly more common in the male nodule group than in the non-nodule group. In females, abdominal obesity, high CRP, hyperglycemia, and hypertension were significantly associated with the detection of thyroid nodules. Logistic regression analysis indicated that gender, age, CRP, hyperglycemia, and smoking history are independent associated factors for the occurrence of thyroid nodules. The detection rate of thyroid nodules among health checkup participants in coastal areas is relatively high, especially among older women, smokers, those with hyperglycemia, and those with high CRP levels. Therefore, it is recommended to strengthen thyroid health management and monitoring for these related population groups.

## 1. Introduction

In recent years, the detection of thyroid nodules in health checkups has become increasingly common, particularly among women. Most of these nodules are benign and asymptomatic, and the proportion of malignant nodules does not exceed 10%. The detection rate through physical examination palpation is only 3 to 7%, but it can reach as high as 70% when high-resolution ultrasound equipment is used.^[1]^ Surveys of adults over the age of 18 show that the detection rate of thyroid nodules in China is as high as 32.4%.^[2]^ The specific causes of thyroid nodules remain unclear, but they are believed to be associated with various factors, including gender, age, lifestyle, height, weight, metabolic abnormalities, radiation exposure, geographical location, and iodine intake levels.^[3–5]^ The detection rate of thyroid nodules varies significantly among different ethnic groups and regions; hence, understanding its epidemiological characteristics, early detection of nodules, and early intervention are crucial for the prevention and treatment of thyroid nodules.

Given the unique environmental and lifestyle factors that residents in coastal areas might face, such as a higher intake of seafood, which could affect the detection rate of thyroid nodules. Qingdao, a coastal city in China, has a higher intake of seafood compared to inland areas. However, although Qingdao is located along the coast, it still belongs to iodine-deficient areas. (The criterion for iodine-deficient areas is a median water iodine content of <40 μg/L, while the median iodine content in our city’s drinking water is only 8.1 μg/L, indicating iodine deficiency in the external environment.) Plants and animals growing in iodine deficient areas are also iodine deficient. This study aims to delve into the detection rate and related factors of thyroid nodules in the eastern coastal area of China through a retrospective analysis of the health checkup population database. The goal of this study is to provide a theoretical basis for the comprehensive prevention and treatment of thyroid nodules, helping to formulate more effective prevention strategies to reduce the impact of thyroid nodules on public health.

## 2. Methods

### 2.1. Participants

A general healthy population from a coastal city in China, examined between January 2013 and December 2022, was included in this study. Inclusion criteria were: Age over 20 years old and first-time visit to this hospital for examination and Able to undergo standardized thyroid ultrasonography. Exclusion criteria included: Individuals with a history of thyroid dysfunction (including thyroid surgery, trauma, hyperthyroidism, or hypothyroidism), Individuals who had undergone thyroid surgery, Individuals with missing information, and Individuals with a history of malignant tumors in other organs or tissues. A total of 15,283 participants were included in the study. Physical examination palpation combined with thyroid ultrasound reports were used to divide participants into nodule and non-nodule groups. This study was reviewed by the Ethics Committee of the People’s Hospital of Chengyang District, Qingdao, and all participants signed an informed consent form.

### 2.2. Basic clinical information collection

This study meticulously recorded participants’ basic clinical information, including age, gender, smoking history, drinking history, and history of chronic diseases such as hypertension and diabetes. Moreover, each participant’s height, weight, and waist circumference (WC) were measured, and body mass index (BMI = weight (kg)/ height (m)^2) was calculated to assess the individual’s weight status.

### 2.3. Determination of biochemical and other indicators

Participants were required to fast for at least 10 hours before blood sampling, which was performed at 8:00 am to measure fasting blood glucose (FBG), total cholesterol (TC), high-density lipoprotein cholesterol (HDL-C), low-density lipoprotein cholesterol (LDL-C), and triglycerides (TG). Liver and kidney functions were also tested. All samples were delivered to the laboratory within 2 hours and analyzed using a fully automatic biochemical analyzer (Roche biochemical analyzer). Additionally, this study collected clinical data and clinical indicators of C-reactive protein (CRP) simultaneously. This study also collected thyroid function indicators, including free thyroxine (FT4), free triiodothyronine (FT3), thyroid-stimulating hormone (TSH), thyroid peroxidase antibody (TPO-Ab), anti-thyroglobulin antibody (Tg-Ab), and TSH receptor antibody (TR).

### 2.4. Thyroid ultrasound examination

All thyroid ultrasound examinations followed the required procedures. Two independent ultrasonographers, each holding at least the rank of attending physician, performed the examinations. Each ultrasonographer completed 2 sets of measurements per patient. The coefficient of variation between the 2 measurements for the same observer was calculated to determine intra-observer repeatability (intra-observer variability). The measurement error range was approximately 1 mm, and the coefficient of variation was <15%. Diagnostic criteria for thyroid nodules included visible hypoechoic solid nodules, cystic nodules, or mixed cystic and solid nodules.

### 2.5. Diagnostic criteria

Hyperglycemia: Defined as abnormal FBG levels according to the “Guideline for the Prevention and Treatment of Type 2 Diabetes in China (2020 Edition).” Dyslipidemia: This study referred to relevant standards and defined dyslipidemia as any of the following conditions: TC ≥ 6.2 mmol/L; TG ≥ 2.3 mmol/L; LDL-C > 4.1 mmol/L; HDL-C < 1.0 mmol/L. Hypertension: Determined according to the “Chinese Guidelines on Prevention and Treatment of Hypertension (2023 Revised Edition).”^[6]^ Hyperuricemia: In a normal purine diet state, fasting blood uric acid levels in men > 420 μmol/L and in women > 360 μmol/L in 2 consecutive measurements were considered hyperuricemia.^[7]^ Overweight and Obesity: Based on BMI, values more than 24 kg/m^2^ were defined as overweight, and <24 kg/m^2^ was defined as normal.^[8]^ Abdominal obesity: WC ≥ 90 cm in men or ≥80 cm in women. Smoking and Drinking Habits: Smoking ≥ 1 cigarette daily for more than one year, or an average of ≥1 drinking occasions per month in the past year. Thyroid nodules: Diagnosed according to the “Expert Consensus on Several Common Clinical Questions of Thyroid and Related Cervical Lymph Node Ultrasound (2018 Edition),”^[9]^ using ultrasound diagnostic criteria to confirm the presence of thyroid nodules.

### 2.6. Statistical methods

This study employed SPSS 25.0 software for data management and statistical analysis. Quantitative data were presented as mean ± standard deviation (±s), and differences between 2 groups were compared using independent sample t-tests. Categorical data were expressed as percentages (%) and compared using chi-square (*χ*^2^) tests. Only participants with complete data were included; the proportion of missing data was <5%. This study is a cross-sectional descriptive analysis without conducting interaction testing. Logistic regression analysis was used to explore the risk factors related to thyroid nodules, using a combination of single-factor and multiple-factor analysis. Each independent variable was separately entered into the univariate model, and variables that are statistically correlated with the outcome (such as *P* < .05) were selected. In conducting a multiple-factor analysis, significant variables from the single-factor analysis and important confounding variables (even if the single factor is not significant) were all included in the model for correction, resulting in adjusted effect values, setting the statistical significance level at *P* < .05.

## 3. Results

### 3.1. General demographic characteristics of the study subjects

This study evaluated a total of 15,283 participants, of whom 91.74% (14,021 individuals) were of Han ethnicity (Table 1). The subjects included 8061 males with a mean age of 45.4 years (range: 20–72 years), and the females with a mean age of 46.8 years (SD = 9.8 years; range: 21–73 years) (Table 2). The overall average BMI of the subjects was 24.62 (SD = ±3.92). Regarding educational levels, 8.40% (1284 individuals) had elementary education or below, 38.45% (5876 individuals) had junior high school education, 31.28% (4781 individuals) had high school education, and 21.87% (3342 individuals) had college education or higher.

**Table 1:** Comparison of clinical data between 2 groups of patients.

Characteristic	Nodule group (n = 4295)	Non-nodule group (n = 10988)	*t* or χ^2^	*P*
Male [*n* (%)]	1731 (40.30)	6330 (57.61)	34.69	.001
Age (years old, $\bar{x}$ ± SD)	51.46 ± 13.44	44.33 ± 12.16	18.36	.003
BMI (kg/m^2^, $\bar{x}$ ± SD)	24.65 ± 3.84	24.59 ± 4.02	1.37	.172
WC (cm, $\bar{x}$ ± SD)	85.27 ± 9.87	81.72 ± 7.91	6.38	.021
SBP (mm Hg, $\bar{x}$ ± SD)	126.52 ± 18.16	127.13 ± 15.53	0.47	.781
DBP (mm Hg, $\bar{x}$ ± SD)	83.34 ± 12.07	75.42 ± 11.98	3.22	.036
TC (mmol/l, $\bar{x}$ ± SD)	5.12 ± 0.92	4.51 ± 1.03	6.53	.001
LDL (mmol/l, $\bar{x}$ ± SD)	2.97 ± 0.76	2.64 ± 0.69	7.14	.001
HDL (mmol/l, $\bar{x}$ ± SD)	1.32 ± 0.57	1.12 ± 0.46	11.20	.001
TG (mmol/l, $\bar{x}$ ± SD)	1.52 ± 0.95	1.49 ± 1.07	0.50	.781
FBG (mmol/l, $\bar{x}$ ± SD)	7.25 ± 1.81	6.45 ± 1.23	4.02	.046
SCR (µmol/l, $\bar{x}$ ± SD)	79.10 ± 14.71	78.13 ± 15.46	0.63	.291
CRP (mg/L, $\bar{x}$ ± SD)	5.79 ± 0.89	4.65 ± 0.73	4.03	.032
UA (µmol/l, $\bar{x}$ ± SD)	317.25 ± 90.62	323.51 ± 84.81	0.82	.624
Smoking [*n* (%)]	1599 (37.23)	2917 (26.55)	19.31	.005
Drinking [*n* (%)]	1949 (45.38)	4576 (41.64)	0.212	.635

BMI = body mass index, BP = blood pressure, CRP = C-reactive protein, FBG = fasting blood glucose, HDL-C = high-density lipoprotein cholesterol, LDL-C = low-density lipoprotein cholesterol, TC = total cholesterol, TG = triglyceride, UA = urea, WC = waist circumference.

**Table 2:** Comparison of detection rates of thyroid nodules.

Age group	Male			Female			Total prevalence rate	*χ* ^2^	*P*
	Sample size	Detection	Detection rate	Sample size	Detection	Detection rate			
20–30	870	70	8.04	872	165	18.92	13.49	15.42	.007
30–40	2043	312	15.27	1830	438	23.93	19.60	28.86	.005
40–50	2782	498	17.90	2770	985	35.56	26.73	92.18	.001
50–60	1335	391	29.28	1237	592	47.86	38.57	61.72	.002
60–70	712	285	40.20	432	317	73.38	56.79	52.78	.005
≥70	315	175	55.55	80	67	83.75	69.65	21.92	.006
Overall	8061	1731	21.47	7221	2564	35.51	28.48	115.79	.002
*χ* ^2^			125.79			98.91	187.32		
*P*			.001			.002	.001		

### 3.2. Clinical information comparison of study subjects

In the comparison between the thyroid nodule group and the non-nodule group, we found that the proportion of females, age, WC, TC, LDL-C, CRP, FBG, diastolic blood pressure, and the proportion of smokers were significantly higher in the thyroid nodule group than in the non-nodule group (*P* < .05) (Table 3). Conversely, the proportion of males and HDL-C were lower in the thyroid nodule group, showing statistical significance (*P* < .05). No statistically significant differences were observed between the 2 groups in TG, systolic blood pressure, serum uric acid, serum creatinine, BMI, or the proportion of drinkers (*P* > .05).

**Table 3:** Analysis of the correlation between different clinical indicators and thyroid nodules in males [cases (%)].

Variables	Nodule group (n = 1731)	Non-nodule group (n = 6330)	*t* or χ^2^	*P*
Overweight and obesity (BMI ≥ 24 kg/m^2^)	1071 (61.87)	3910 (61.77)	0.92	.815
Abdominal obesity (WC ≥ 90cm)	890 (51.41)	3161 (49.94)	0.61	.721
Hyperglycemia	340 (19.64)	563 (8.91)	12.35	.022
Hypertension	775 (44.77)	1410 (22.27)	78.35	.001
High TC	640 (36.97)	2280 (36.02)	0.22	.526
High LDL	418 (24.15)	1562 (24.68)	0.53	.590
Low HDL	483 (27.90)	1728 (27.29)	−0.76	.452
High TG	823 (47.54)	3005 (47.47)	1.20	.098
Hyperuricemia	339 (19.58)	1263 (19.95)	0.46	<683
High-sensitive CRP	277 (16.00)	551 (8.70)	23.47	.003
Smoking	1131 (65.34)	2946 (46.82)	51.28	.001
Drinking	1219 (70.42)	4557 (71.99)	0.12	.254

BMI = body mass index, BP = blood pressure, CRP = C-reactive protein, FBG = fasting blood glucose, HDL-C = high-density lipoprotein cholesterol, LDL-C = low-density lipoprotein cholesterol, TC = total cholesterol, TG = triglyceride, UA = urea, WC = waist circumference.

### 3.3. Impact of age and gender on the detection rate of thyroid nodules

In this study, among the 15,283 people examined, 4295 cases of thyroid nodules were discovered, resulting in an overall detection rate of 28.48%, while the detection rate was 35.51% in females, which was higher than that in males (21.47%) (Table 2). Specifically, the detection rate of thyroid nodules in females was 35.51%, significantly higher than that in males at 21.47% (*χ*^2^ = 115.79, *P* = .002), indicating a significant statistical difference in gender for the occurrence of thyroid nodules. Further analysis by age groups revealed that, regardless of gender, the detection rate of thyroid nodules showed an increasing trend with age. In all age groups, the detection rate was higher in females than in males, with statistically significant differences (all *P* < .01)..

### 3.4. Analysis of clinical indicators related to thyroid nodules in males

Among males, the proportions of elevated CRP, hyperglycemia, hypertension, and smoking history were significantly higher in the nodule group than in the non-nodule group (*P* < .05) (Table 3). This suggests that these factors are associated with the occurrence of thyroid nodules. However, there were no statistically significant differences between the 2 groups in terms of overweight and obesity (BMI ≥ 24.0 kg/m^2^), abdominal obesity, high TC, high LDL-C, low HDL-C, high TG, high uric acid levels, and history of drinking (*P* > .05).

### 3.5. Analysis of clinical indicators related to thyroid nodules in females

The analysis of female participants revealed some significant differences between the thyroid nodule group and the non-nodule group. Notably, the proportion of abdominal obesity was significantly higher in the thyroid nodule group (*P* < .05), whereas there was no significant difference in the distribution of overweight and obesity between the 2 groups (*P* > .05) (Table 4). Additionally, the proportions of high CRP, hyperglycemia, hypertension, increased TC levels, increased LDL-C levels, and decreased HDL-C levels were significantly higher in the nodule group (*P* < .05). However, there were no statistically significant differences in high TG, high uric acid levels, smoking history, and drinking history between the 2 groups (*P* > .05).

**Table 4:** Analysis of the correlation between different clinical indicators and thyroid nodules in females [cases (%)].

	Nodule group (n = 2564)	Non-nodule group (n = 4657)	*t* or χ^2^	*P*
Overweight and obesity (BMI ≥ 23 kg/m^2^)	1284 (50.08)	2379 (51.08)	1.16	.069
Abdominal obesity (WC ≥ 80cm)	1712 (66.77)	2067 (44.38)	5.09	.025
Hyperglycemia	250 (9.75)	205 (4.40)	3.17	.045
Hypertension	747 (29.13)	890 (19.11)	14.78	.001
High TC	1011 (39.43)	1418 (30.45)	3.68	.047
High LDL	741 (28.90)	972 (20.87)	2.35	.039
Low HDL	200 (7.81)	180 (3.86)	−2.24	.042
High TG	750 (29.25)	824 (17.69)	1.00	.097
Hyperuricemia	139 (5.42)	236 (5.07)	0.51	.621
High-sensitive CRP	218 (8.50)	173 (3.71)	6.47	.035
Smoking	17 (0.06)	6 (0.06)	1.02	.083
Drinking	130 (5.07)	258 (5.54)	0.92	.735

BMI = body mass index, BP = blood pressure, CRP = C-reactive protein, FBG = fasting blood glucose, HDL-C = high-density lipoprotein cholesterol, LDL-C = low-density lipoprotein cholesterol, TC = total cholesterol, TG = triglyceride, UA = urea, WC = waist circumference.

### 3.6. Logistic regression analysis of risk factors related to thyroid nodules

A logistic regression analysis was conducted to explore factors associated with thyroid nodules. The analysis used the presence of thyroid nodules as the dependent variable (present = 1, absent = 0) and gender (male = 0, female = 1), age, CRP levels (normal = 0, high = 1), WC (male < 90 cm or female < 80 cm = 0, male ≥ 90 cm or female ≥ 80 cm = 1), BMI (<24.0 kg/m^2^ = 0, ≥24.0 kg/m^2^ = 1), blood sugar (normal = 0, high = 1), blood pressure (normal = 0, high = 1), TG (normal = 0, high = 1), TC (normal = 0, high = 1), LDL-C (normal = 0, high = 1), HDL-C (normal = 0, low = 1), uric acid (normal = 0, high = 1), smoking status (none = 0, yes = 1), and alcohol consumption habits (none = 0, yes = 1) as independent variables. The analysis results, as shown in Table 5, indicate that gender, age, CRP levels, blood sugar levels, and smoking history are significant risk factors for the occurrence of thyroid nodules (*P* < .05). Specifically, compared to males, females have a 3.129 times higher risk of developing thyroid nodules; for every 10-year increase in age, the risk of disease increases by 1.482 times; individuals with high CRP levels have a 1.62 times higher risk of disease than those with normal CRP levels; people with hyperglycemia have a 1.258 times higher risk than those with normal blood sugar levels; smokers have a 2.217 times higher risk than nonsmokers. However, BMI (BMI ≥ 23.0), WC (male ≥ 90 cm or female ≥ 80 cm), and hypertension did not show statistically significant correlations as risk factors (*P* > .05).

**Table 5:** Analysis of risk factors related to thyroid nodules.

Variables	β	Wald	Odds ratio	95% CI	*P*
Gender	1.236	92.54	3.129	2.622–4.465	.001
Age	0.729	77.74	1.482	1.216–1.836	.012
Smoking	0.339	14.290	2.217	1.557–2.887	.001
Drinking	0.039	0.97	1.129	0.887–1.362	.739
UA	0.050	0.330	1.050	0.894–1.244	.563
CRP	0.358	8.919	1.620	1.271–2.193	.001
BMI	0.129	1.74	1.063	0.845–1.321	.175
WC	0.080	0.250	1.080	0.802–1.471	.621
BG	0.295	4.74	1.258	1.035–1.711	.031
BP	0.030	1.327	1.117	0.821–1.819	.814
TC	0.380	0.250	1.080	0.802–1.471	.621
LDL-C	0.080	1.150	1.080	0.943–1.252	.282
HDL	−0.040	0.210	0.960	0.822–1.131	.650
TG	0.060	0.370	0.950	0.793–1.130	.541

BMI = body mass index, BP = blood pressure, CRP = C-reactive protein, FBG = fasting blood glucose, HDL-C = high-density lipoprotein cholesterol, LDL-C = low-density lipoprotein cholesterol, TC = total cholesterol, TG = triglyceride, UA = urea, WC = waist circumference.

## 4. Discussion

The thyroid, as a crucial endocrine organ in the human body, can cause various discomforts due to abnormalities in its structure and function. Thyroid nodules, which are masses formed by the abnormal proliferation of thyroid cells, most often do not cause any discomfort.^[10,11]^ However, in rare cases, they can lead to a sensation of a foreign body in the throat or slight swelling pain, often resulting in diagnostic delays. With advances in medical diagnostic technologies, the detection rate of thyroid nodules has significantly increased. Studies have shown that the detection rate of thyroid nodules varies significantly across different regions, with about 20 to 30% in Northern Europe, over 65% in North America, and even more significant differences within various regions of China. Our research found that the detection rate of thyroid nodules in this coastal region is 28.48%, which is at a moderate level nationally.^[12]^

The mechanism behind the occurrence of thyroid nodules is complex and involves many factors, including psychological stress, exposure to radioactive materials, excessive iodine intake, smoking history, and pathogen infections.^[13–15]^ Our findings reveal that smoking is a significant risk factor for thyroid nodules in men, but not in women, possibly due to the relatively lower proportion of female smokers. Additionally, alcohol consumption does not seem to have a direct correlation with the occurrence of thyroid nodules.^[16,17]^

In recent years, with the improvement of living standards and the acceleration of life’s pace, chronic metabolic diseases such as overweight, obesity, dyslipidemia, hypertension, hyperglycemia, and hyperuricemia have become increasingly common.^[18,19]^ Although there are studies on the relationship between these metabolic abnormalities and thyroid nodules, the conclusions are inconsistent. Our study shows that dyslipidemia is related to thyroid nodules in women, but no such association was found in men.^[20]^

Univariate and multivariate analyses revealed that age, sex, elevated CRP, and hyperglycemia were associated with the detection of thyroid nodules. Female sex showed a 3.129-fold higher association with thyroid nodule detection compared with male sex. For every 10-year increase in age, the association with thyroid nodule detection increased by a factor of 1.53, which is consistent with previous findings.^[18]^ In the present study, patients with elevated CRP levels had a 1.62-fold higher association with thyroid nodule detection than those without elevated CRP. CRP is a sensitive marker of systemic inflammation and is involved in the initiation and progression of inflammatory responses, making it useful for predicting disease development and outcomes. Inflammatory processes can disrupt the local architecture of the thyroid gland, leading to pulsatile increases in TSH, thereby promoting the formation of thyroid nodules. Hyperglycemia was associated with a 1.258-fold higher likelihood of thyroid nodule detection compared with normoglycemia. A potential explanation is that some patients with diabetes mellitus receive exogenous insulin therapy due to insulin resistance or insulin deficiency, resulting in elevated serum insulin levels. Insulin, acting as a thyroid growth factor, may stimulate thyroid cell proliferation, thereby increasing the risk of thyroid hyperplasia and nodule formation.^[19]^ Although previous studies have reported risk factor analyses indicating that hyperglycemia has no significant effect on thyroid nodules but that hypertension is a risk factor for thyroid nodules, such an association was not observed in the present study.^[20–22]^

It is noteworthy that, although this study has revealed significant correlations between factors such as smoking, high CRP levels, and hyperglycemia with the risk of thyroid nodules, it also has certain limitations, such as an imbalance in the geographical distribution of samples.^[21]^ Therefore, in annual health checkups, especially for related population groups such as the elderly, females, individuals with a history of smoking, hyperglycemia, and high CRP levels, screening for thyroid diseases should be intensified, including thyroid ultrasound examinations, to achieve early detection, diagnosis, and intervention.^[22]^

In summary, this study not only provides the detection rate of thyroid nodules in a healthy population in this coastal area and its correlation with various clinical indicators but also offers a theoretical basis for the comprehensive management of thyroid nodules. Future research needs to further explore the mechanisms behind thyroid nodule development.

Several limitations should be acknowledged. First, the cross-sectional design precludes causal inferences. Second, selection bias may exist, as only individuals who voluntarily underwent health examinations were included. Third, residual confounding may persist due to unmeasured variables. Fourth, iodine intake, a key factor given the coastal setting, was not assessed. Finally, temporal variations over the 10-year study period may have influenced the results.

## Acknowledgments

We thank you all the participants for information donation.

## Author contributions

**Conceptualization:** Xuemei Sun, Pei Wang, Xiaopang Rao.

**Data curation:** Xuemei Sun, Xiaopang Rao.

**Formal analysis:** Xuemei Sun, Yu Zhang, Xiaopang Rao.

**Investigation:** Yu Zhang.

**Methodology:** Yu Zhang.

**Project administration:** Yu Zhang.

**Resources:** Yu Zhang.

**Writing – original draft:** Xuemei Sun, Pei Wang.

**Writing – review & editing:** Xiaopang Rao.
